# Two Cases of Intraductal Papilloma with Sebaceous Metaplasia of the Breast

**DOI:** 10.70352/scrj.cr.25-0658

**Published:** 2026-03-13

**Authors:** Kurumi Okada, Takashi Tashiro, Toshitaka Okuno, Sachiko Yuen, Nao Yamamoto, Yoichiro Kuwata, Naoki Sugiyama, Nobuyuki Saga, Maki Kanzawa, Hajime Matsumoto, Kazuhiko Yamagami

**Affiliations:** 1 Department of Breast Surgery and Oncology, Shinko Hospital, Kobe, Hyogo, Japan; 2 Department of Breast Surgery, Kobe City Nishi-Kobe Medical Center, Kobe, Hyogo, Japan; 3 Department of Pathology, Shinko Hospital, Kobe, Hyogo, Japan; 4 Department of Diagnostic Radiology, Kobe City Nishi-Kobe Medical Center, Kobe, Hyogo, Japan; 5 Department of Pathology, Kobe City Nishi-Kobe Medical Center, Kobe, Hyogo, Japan

**Keywords:** breast, sebaceous metaplasia, intraductal papilloma

## Abstract

**INTRODUCTION:**

Intraductal papilloma is a common benign breast lesion that typically presents with nipple discharge or a palpable mass. While apocrine metaplasia is frequently observed, sebaceous metaplasia is very rare in breast lesions, with only a few cases reported to date. We herein describe two surgically resected cases of intraductal papilloma with sebaceous metaplasia, both of which were histologically proven benign following imaging findings that were suspicious for malignancy.

**CASE PRESENTATION:**

Case 1: A 47-year-old woman presented with a left breast mass detected during routine screening. Imaging revealed an intracystic lesion with mural nodules and features that were suspicious for malignancy. Surgical excision was performed, and a histopathological examination confirmed intraductal papilloma with sebaceous metaplasia. Case 2: A subareolar mass was detected in a 62-year-old woman during screening ultrasonography. Cytology showed atypical features suspicious for malignancy. Surgical resection yielded a diagnosis of intraductal papilloma with sebaceous metaplasia.

**CONCLUSIONS:**

These cases represent rare occurrences of intraductal papilloma with sebaceous metaplasia, an entity for which only one case has been reported in the English literature and lacked imaging documentation. Our results highlight characteristic imaging features, such as cyst wall thickening and solid internal components with marked enhancement, which may mimic malignancy. These findings provided new insights into the imaging and pathological spectrum of metaplastic changes in intraductal papilloma. Recognition that sebaceous metaplasia can occur within an otherwise benign papilloma may help avoid overinterpretation of suspicious imaging findings and contribute to more appropriate clinical decision-making.

## Abbreviations

IP
: intraductal papilloma
US
: ultrasonography

## INTRODUCTION

IP is a benign epithelial tumor of the breast that commonly presents with nipple discharge or a palpable mass.^1,2)^ Apocrine metaplasia is a frequent histological finding in IP, whereas sebaceous metaplasia is extremely rare. To the best of our knowledge, only one case of IP with sebaceous metaplasia has been reported in the English literature to date and lacked imaging documentation.^3,4)^

We herein describe two surgically resected cases of IP with histologically confirmed sebaceous metaplasia. Both cases were initially suspected to be malignant based on imaging or cytology, underscoring the diagnostic challenges posed by this unusual histological feature.

## CASE PRESENTATION

### Case 1

A 47-year-old woman was referred for the evaluation of a mass in the left breast detected during routine screening. A physical examination revealed a firm, non-tender mass in the mid-outer quadrant of the left breast. There was no nipple discharge or skin change.

**Fig. 1:**
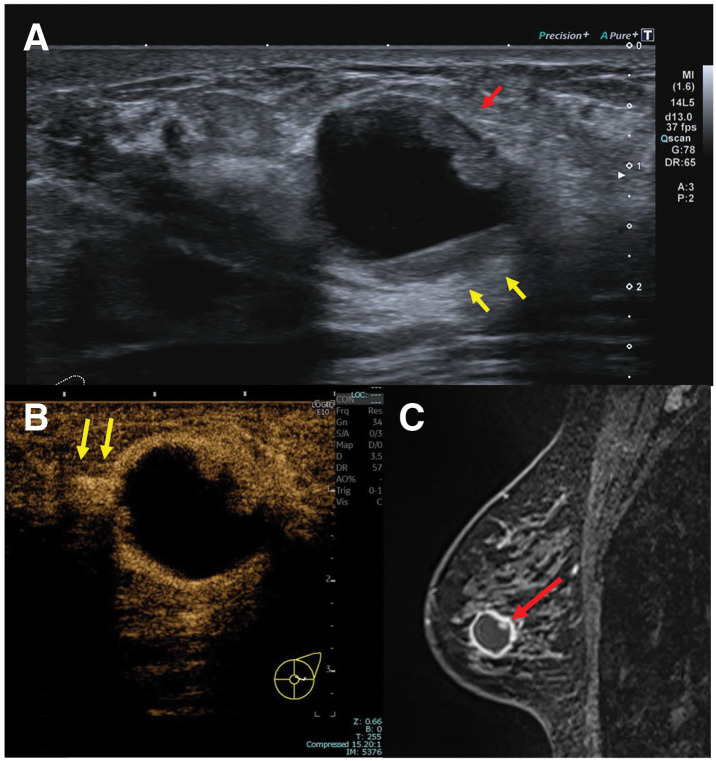
Imaging findings of Case 1. (**A**) B-mode US demonstrates a 20-mm intracystic mass with broad-based mural nodules (red arrow) and internal fluid–fluid levels (yellow arrows). (**B**) Contrast-enhanced US shows homogeneous enhancement of the cyst wall and mural nodules, with enhancement extending towards the nipple (yellow arrows) corresponding to the hypoechoic area on B-mode US. (**C**) MRI shows a cystic tumor with a homogeneously enhanced thickened wall and mural nodules (red arrow), consistent with the US findings. US, ultrasonography

Mammography showed a mass with indistinct margin. US (Aplio a550; Canon Medical Systems, Tochigi, Japan) revealed an intracystic mass measuring 20 × 19 × 16 mm with broad-based mural nodules and internal fluid–fluid levels (**Fig. 1A**). Color Doppler US showed mild vascularity within the solid components. Homogeneous enhancement of the cyst wall and mural nodules was noted on contrast-enhanced US (LOGIQ E10; GE HealthCare, Tokyo, Japan). A hypoechoic area extending toward the nipple exhibited low vascularity with weak contrast enhancement (**Fig. 1B**). The early dynamic images on breast MRI (Vida, 3T; Siemens Healthineers, Tokyo, Japan) demonstrated thick homogeneous enhancement of the cyst wall and mural nodules, which raised suspicion for intracystic carcinoma (**Fig. 1C**).

Fine-needle aspiration cytology revealed benign epithelial cell clusters without nuclear atypia, resulting in a diagnostic discrepancy between cytology and imaging. Core-needle biopsy or vacuum-assisted biopsy was considered to carry a risk of disrupting the cystic structure, potentially leading to leakage of cyst contents and concern for tumor cell dissemination. Therefore, the option of surgical excision was explained, and segmental excision of the breast was performed for a definitive diagnosis.

**Fig. 2:**
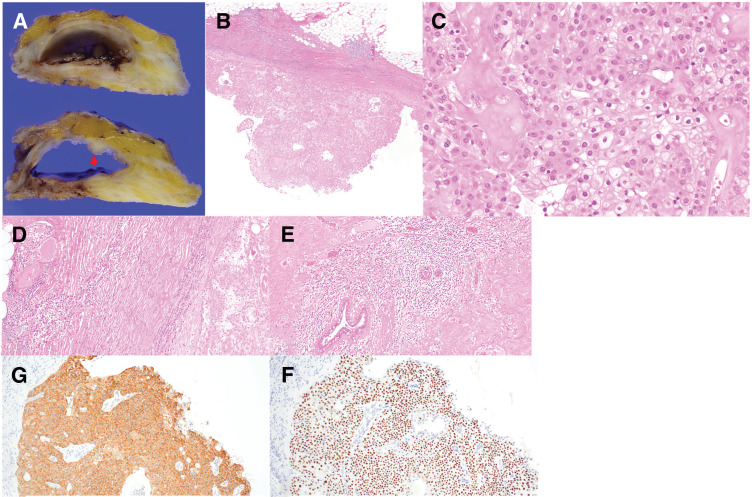
Histopathological findings of Case 1. (**A**) Gross examination shows a cyst with a thickened wall and a mural nodule (red arrow). (**B**, **C**) H&E staining reveals proliferating epithelial cells with foamy cytoplasm and abundant intracellular lipid droplets, exhibiting a lipid-rich morphology. (**D**) Prominent infiltration of inflammatory cells is observed around the lesion and (**E**) in the periductal area adjacent to the nipple. (**F**) Immunohistochemical staining shows positive reactivity for p63 and (**G**) CK5/6 in the proliferating cells. H&E, hematoxylin–eosin

A gross examination of the resected specimen revealed a cyst with a thickened wall and a mural nodule (**Fig. 2A**). Histologically, the lesion was composed of the biphasic epithelial proliferation of luminal and abluminal cells around fibrovascular cores, consistent with IP (**Fig. 2B**). Some luminal cells had a foamy cytoplasm, abundant intracellular lipid droplets, and a lipid-rich morphology distinct from normal mammary epithelial cells (**Fig. 2C**), which are features that are consistent with sebaceous metaplasia as previously described in the literature.^3)^ In addition, prominent infiltration of inflammatory cells was observed in the perilesional area and within the periductal region adjacent to the nipple. These inflammatory changes likely accounted for contrast enhancement extending toward the nipple on contrast-enhanced US as well as circumferential wall enhancement on both contrast-enhanced US and MRI (**Fig. 2D** and **2E**). Immunohistochemical staining revealed that these cells were positive for p63 and cytokeratin 5/6 (**Fig. 2F** and **2G**) and negative for estrogen receptor (ER) and androgen receptor, supporting sebaceous metaplasia.

Based on morphological and immunohistochemical findings, the final diagnosis was IP with sebaceous metaplasia.

### Case 2

A 62-year-old woman underwent screening for US, which revealed a small hypoechoic mass in the subareolar region of the left breast. A physical examination detected a firm, non-tender mass without nipple discharge or skin changes.

**Fig. 3:**
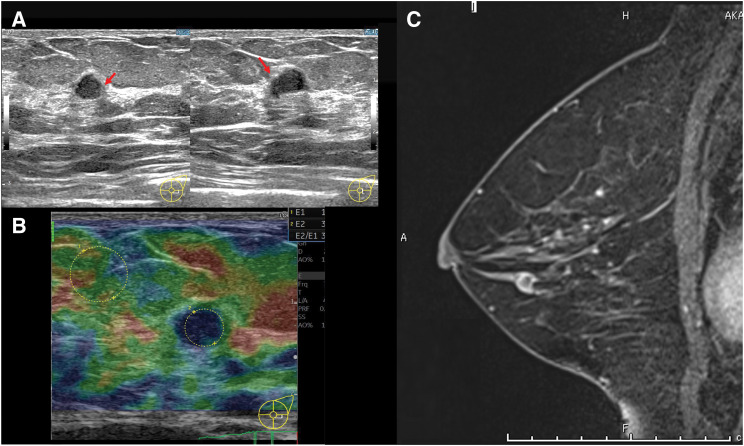
Imaging findings of Case 2. (**A**) B-mode US demonstrates a 7-mm cystic mass with a papillary internal component (arrows) and (**B**) a Tsukuba elasticity score of 4. (**C**) MRI shows a small cystic tumor with a markedly enhanced thickened wall and heterogeneous internal enhancement. US, ultrasonography

Mammography showed a small focal asymmetry in the medial inferior quadrant of the left breast. US (LOGIC E10) showed a hypoechoic mass measuring 7.8 × 6.2 × 5.0 mm in the lower outer quadrant of the left breast, which was recognized as a mixed lesion containing a papillary component on closer observation (**Fig. 3A**). Elastography according to the Tsukuba elasticity score classified the lesion as score 4^5)^ (**Fig. 3B**), reflecting increased tissue stiffness consistent with a potentially malignant process. The early dynamic images on breast MRI (Skyra, 3T; Siemens Healthineers) showed a thickened cyst wall with marked enhancement and heterogeneous internal enhancement (**Fig. 3C**). These imaging characteristics are uncommon in small breast tumors and further suggested the possibility of malignancy.

Fine-needle aspiration cytology revealed loosely cohesive clusters of luminal cells with a foamy cytoplasm. Some luminal cells had prominent nucleoli and intracytoplasmic lumina, suggestive of atypia. The lesion was categorized as suspicious for malignancy. Core needle biopsy revealed papillary epithelial proliferation with a foamy cytoplasm and numerous intracellular lipid droplets, which were morphologically distinct from the normal mammary epithelium. Immunohistochemical staining showed positivity for CK5/6 and focal low expression of ER and progesterone receptors. Mucicarmine staining confirmed the presence of intracytoplasmic lumina, a feature known to be associated with increased malignant potential in papillary breast lesions.^2)^ Based on the imaging and cytological findings, surgical excision was therefore selected to obtain a definitive diagnosis rather than short-term imaging follow-up. Although the lesion was small (7 mm), both US and MRI demonstrated features strongly suggestive of malignancy, and cytologic atypia identified by mucicarmine staining further supported the decision to proceed with surgical excision.

**Fig. 4:**
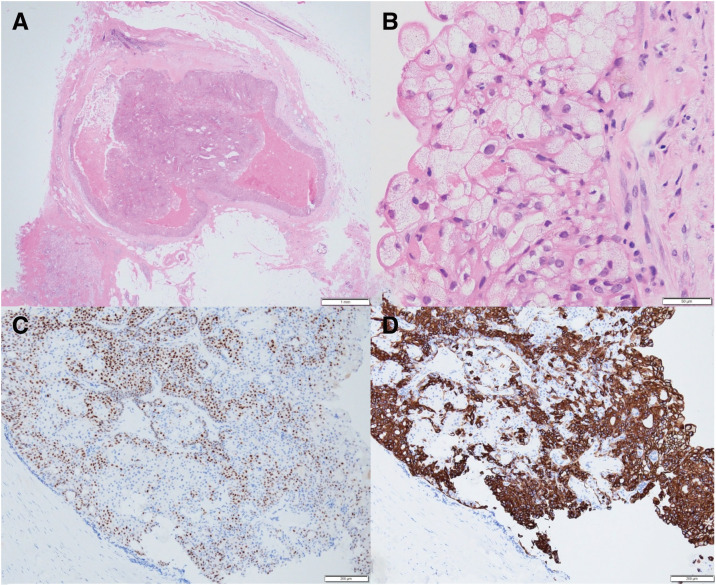
Histopathological findings of Case 2. (**A**) H&E staining shows a cystic tumor with papillary to solid epithelial proliferation and a thickened fibrous cyst wall, and (**B**) sebaceous gland differentiation within the internal papillary proliferation. (**C**) Immunohistochemical analysis shows positivity for p63 and (**D**) CK5/6 in the sebaceous glandular area. H&E, hematoxylin–eosin

A histological examination of the excised specimen revealed papillary to solid epithelial proliferation within a dilated duct, with the thickened cyst wall composed of fibrous tissue accompanied by edema (**Fig. 4A**). Luminal cells had a foamy cytoplasm and abundant lipid droplets (**Fig. 4B**). No cytological signs of malignancy, such as nuclear atypia, increased mitoses, or invasion, were identified. Immunohistochemistry confirmed positivity for CK5/6 and p63 (**Fig. 4C** and **4D**), with focal ER positivity.

Based on these results, the final diagnosis was IP with sebaceous metaplasia.

## DISCUSSION

IP is a benign epithelial lesion characterized by papillary structures with a central fibrovascular core. It typically maintains a dual-layered architecture consisting of luminal and myoepithelial cells.^1,2)^ Central IP often presents with unilateral serous or bloody nipple discharge, whereas peripheral IP is frequently asymptomatic and incidentally detected on imaging.

IP commonly appears as well-defined cystic lesions with internal papillary projections on US, and the presence of a single vascular stalk on color Doppler is generally suggestive of benignity. MRI findings vary depending on lesion size and complexity; smaller lesions generally have smooth margins and homogeneous enhancement, whereas larger or complex lesions may exhibit irregular margins or a heterogeneous internal architecture.

Apocrine metaplasia is a well-known histological feature of IP and may be accompanied by the focal loss of myoepithelial cells. Squamous metaplasia is rarely observed, typically arising in the context of infarction or cystic degeneration, and is considered to result from localized ischemia, inflammation, or mechanical stimuli. Sebaceous metaplasia is an extremely rare finding in IP; only one case has been reported in the English literature and lacked imaging documentation.^3,4)^

Histologically, sebaceous cells are characterized by a foamy cytoplasm, lipid droplets, and distinct cell borders, features that markedly differ from the normal mammary epithelium.^6–8)^ Sebaceous differentiation in cutaneous adnexal tissues is considered to be physiological^6)^ as well as apocrine and squamous differentiation, and given the shared ectodermal origin of the breast and skin, sebaceous differentiation within the breast epithelium may be embryologically plausible. Experimental studies using mammary epithelial cell lines demonstrated that the transfection of metastatic rat mammary tumor DNA (MTLn3) into normal mammary epithelial cells (Rama 37) induced squamous differentiation accompanied by sebaceous-like features, suggesting a differentiation pathway from squamous to sebaceous metaplasia.^4)^ Taken together, sebaceous metaplasia appears analogous to apocrine or squamous metaplasia and is likely to represent a benign reactive differentiation process, potentially associated with chronic inflammation, ischemia, or local microenvironmental changes. These observations support the concept that sebaceous metaplasia in IP is not a premalignant condition but rather a benign metaplastic alteration.

Sebaceous differentiation in human breast pathology, including the two cases presented herein, has only been reported in 9 cases of adenoid cystic carcinoma, 6 cases of ductal carcinoma, 5 cases of adenomyoepithelioma, and 4 cases of benign IP.^4,9)^ None of these reports described sebaceous metaplasia itself as exhibiting malignant behavior or acting as a premalignant lesion; rather, sebaceous differentiation has consistently been regarded as an incidental or reactive phenomenon within the underlying tumor. Accordingly, the presence of sebaceous metaplasia alone should not be regarded as an indication for aggressive surgical management in the absence of other histological or clinical features suggestive of malignancy.

Both of our cases showed imaging features suggestive of malignancy on US and MRI. In Case 1, suspicious features on US and MRI included cyst wall thickening and strong contrast enhancement, fluid–fluid levels, and the extension of a hypoechoic area toward the nipple. Case 2 showed a cystic mass with a papillary internal component on US and thickened strong wall enhancement on contrast-enhanced MRI. Notably, the degree of cyst wall thickening was disproportionate to the small lesion size, contributing to the malignant impression.

These imaging features, that is, marked enhancement of the thickened wall and mural nodules, corresponded well with the histological findings of thickened fibrous cyst wall accompanied by inflammation or edema and papillary to solid epithelial proliferation. We speculate that these imaging characteristics might be related to sebaceous metaplasia in IP. On the other hand, these imaging characteristics should not be regarded as specific to sebaceous metaplasia. Similar imaging features have been reported in IP with apocrine metaplasia and in intracystic carcinoma.^10,11)^ Accordingly, preoperative differentiation of IP with sebaceous metaplasia from intracystic carcinoma remains challenging, and imaging findings alone are insufficient for definitive discrimination.

Awareness of this rare histological variant of IP is essential for accurate diagnostic interpretation and informed clinical decision-making. Importantly, recognition that sebaceous metaplasia can occur within an otherwise benign papilloma may help avoid overinterpretation of suspicious imaging findings and unnecessary surgical excision, and supports consideration of conservative management with imaging surveillance in appropriately selected cases through multidisciplinary integration of radiologic, pathologic, and clinical findings.

## CONCLUSIONS

Sebaceous metaplasia in IP is an extremely rare histological finding and its key cytological features include a foamy cytoplasm, lipid droplet accumulation, and a sharply demarcated sebaceous-like morphology.

This is the first report of presenting the imaging features of IP with sebaceous metaplasia. The imaging features of IP with sebaceous metaplasia on US and MRI mimicked malignancy, where cyst wall thickening and solid internal components with marked enhancement were consistent with the histological findings of thickened fibrous cyst wall accompanied by inflammation or edema and papillary to solid epithelial proliferation.

Awareness of this rare histological variant of IP is essential for accurate diagnostic interpretation and informed clinical decision-making. Recognizing that sebaceous metaplasia can occur as part of an otherwise benign papilloma may help avoid unnecessary surgical intervention in appropriately selected cases.

## DECLARATIONS

### Availability of data and materials

All data generated or analyzed during this study are included in the published article.
